# Genetic control of *longissimus dorsi* muscle gene expression variation and joint analysis with phenotypic quantitative trait loci in pigs

**DOI:** 10.1186/s12864-018-5386-2

**Published:** 2019-01-03

**Authors:** Deborah Velez-Irizarry, Sebastian Casiro, Kaitlyn R. Daza, Ronald O. Bates, Nancy E. Raney, Juan P. Steibel, Catherine W. Ernst

**Affiliations:** 1 Department of Animal Science, Michigan State University, East Lansing, MI 48824 USA; 2 Department of Fisheries and Wildlife, Michigan State University, East Lansing, MI 48824 USA

**Keywords:** eQTL, Skeletal muscle, RNA-Seq, Transcriptome, Pig

## Abstract

**Background:**

Economically important growth and meat quality traits in pigs are controlled by cascading molecular events occurring during development and continuing throughout the conversion of muscle to meat. However, little is known about the genes and molecular mechanisms involved in this process. Evaluating transcriptomic profiles of skeletal muscle during the initial steps leading to the conversion of muscle to meat can identify key regulators of polygenic phenotypes. In addition, mapping transcript abundance through genome-wide association analysis using high-density marker genotypes allows identification of genomic regions that control gene expression, referred to as expression quantitative trait loci (eQTL). In this study, we perform eQTL analyses to identify potential candidate genes and molecular markers regulating growth and meat quality traits in pigs.

**Results:**

Messenger RNA transcripts obtained with RNA-seq of *longissimus dorsi* muscle from 168 F2 animals from a Duroc x Pietrain pig resource population were used to estimate gene expression variation subject to genetic control by mapping eQTL. A total of 339 eQTL were mapped (FDR ≤ 0.01) with 191 exhibiting local-acting regulation. Joint analysis of eQTL with phenotypic QTL (pQTL) segregating in our population revealed 16 genes significantly associated with 21 pQTL for meat quality, carcass composition and growth traits. Ten of these pQTL were for meat quality phenotypes that co-localized with one eQTL on SSC2 (8.8-Mb region) and 11 eQTL on SSC15 (121-Mb region). Biological processes identified for co-localized eQTL genes include calcium signaling (*FERM*, *MRLN*, *PKP2* and *CHRNA9*), energy metabolism (*SUCLG2* and *PFKFB3*) and redox hemostasis (*NQO1* and *CEP128*), and results support an important role for activation of the PI3K-Akt-mTOR signaling pathway during the initial conversion of muscle to meat.

**Conclusion:**

Co-localization of eQTL with pQTL identified molecular markers significantly associated with both economically important phenotypes and gene transcript abundance. This study reveals candidate genes contributing to variation in pig production traits, and provides new knowledge regarding the genetic architecture of meat quality phenotypes.

**Electronic supplementary material:**

The online version of this article (10.1186/s12864-018-5386-2) contains supplementary material, which is available to authorized users.

## Background

Genomic improvement techniques have significantly advanced livestock breeding in recent years. Genomic regions harboring single nucleotide polymorphisms (SNP) accounting for a significant portion of phenotypic variation for economically important traits have been identified and implemented in marker assisted selection [1–3]. In pigs, these efforts have identified candidate genes affecting meat quality (e.g., *CRC1*, *PRKAG3*, *CAST*), weight gain (e.g., *MC4R*) and litter size (e.g., *ESR*) [4]. However, we still do not fully understand the molecular mechanisms underlying the variability observed in pork traits.

Meat quality traits are highly correlated. During the conversion of muscle to meat, Ca^2+^ ions are released from the sarcoplasmic reticulum and the anaerobic production of ATP leads to the accumulation of lactic acid that reduces muscle pH [5]. The rate of pH decline and release of Ca^2+^ directly influences water holding capacity, meat color and the rate of proteolytic activity that leads to meat tenderization [5]. While these molecular processes have been extensively studied with numerous QTL identified for tenderness, drip loss, pH, meat color and enzyme activity [6], we know little of the genetic architecture regulating these traits. This is likely due to the high variability of meat quality traits that are known to be heavily influenced by both genetic and environmental factors such as antemortem handling [7–9]. Regulators of gene expression have been used to study the molecular bases of polygenetic phenotypic differences in swine populations [10–13]. In addition, expression quantitative trait loci (eQTL) maps provide a foundation to study divergent molecular processes in livestock species [2, 14]. This approach has been successful in identifying candidate genes, causative variants and molecular networks regulating phenotypic traits in swine, including back fat [15], drip loss [16], glycolytic potential [13], plasma cortisol levels [10] and lipid metabolism [17].

For meat quality traits, cascading molecular events starting before exsanguination and continuing throughout the conversion of muscle to meat play a critical role in determining the eating quality of pork. By studying the transcriptomic profile of the initial steps leading to the conversion of muscle to meat, we can elucidate key regulators of polygenetic trait phenotypes. Specifically, we can identify gene transcripts subject to genetic control that potentially regulate complex traits by mapping eQTL and testing their co-localization with phenotypic QTL (pQTL).

In this study, we use an F2 Duroc x Pietrain resource population developed at Michigan State University [18, 19] (the MSUPRP) to map eQTL for *longissimus dorsi* muscle in order to identify local and distant regulators of transcript abundance, and to estimate narrow-sense heritability (h^2^) of gene expression. Putative hotspots are also of interest where a single marker is associated with the expression of multiple genes, serving as a potential master regulator that can account for a significant portion of phenotypic variation. A co-localization analysis of eQTL with pQTL reveals novel insights into the genetic architecture of meat quality, carcass composition and growth traits.

## Results

### Identification of eQTL

A genome wide association study (GWAS) was conducted using 23,162 SNP markers and 15,249 transcript abundance profiles for 168 F2 pigs. The GWAS identified 339 eQTL (3094 significant gene marker associations; whole genome FDR ≤ 0.01 per gene) for 321 gene transcripts and 2523 molecular markers (Additional file 1 Table S1). The number of SNP associated with an eQTL was on average 9.09 ± 15.07, and the size of each eQTL interval was on average 11.59 ± 22.30-Mb (Table 1). A total of five eQTL intervals contained an additional peak determined through conditional analysis by fixing the peak eQTL SNP. These eQTL intervals were observed on SSC1, 10 and 12 (entries in Table S1 with the same transcript identifiers).

**Table 1:** eQTL summary among regulator types

Gene Regulator	N^a^	Min^b^	Max^c^	Mean^d^	SD^e^
Average length of eQTL interval ^f^					
All regulators	339	0	175.19	11.59	22.30
Local	168	0	175.19	21.62	27.59
Plausible Local	23	0	11.44	1.36	2.77
Distant Same Chromosome	61	0	25.55	2.22	5.46
Distant	87	0	69.76	1.47	7.92
Average distance from eQTL to gene transcript position ^f^					
Total Same Chromosome	252	4.11e-4	104.75	3.65	12.15
Local	168	4.11e-4	25.07	1.90	3.86
Plausible Local	23	4.28e-3	1.49	0.20	0.40
Distant Same Chromosome	61	3.73e-3	104.75	9.75	22.92
Distant	87	–	–	–	–
Number of SNP associations per eQTL					
All regulators	339	1	105	9.09	15.07
Local	168	1	105	16.45	18.65
Plausible Local	23	1	14	2.87	3.08
Distant Same Chromosome	61	1	17	2.05	2.37
Distant	87	1	5	1.46	0.97
Heritability estimates					
All regulators	339	5.47e-10	0.97	0.32	0.23
Local	168	5.47e-10	0.97	0.42	0.22
Plausible Local	23	0.04	0.63	0.32	0.16
Distant Same Chromosome	61	1.19e-09	0.78	0.29	0.23
Distant	87	1.34e-09	0.76	0.17	0.17

^a^Number of eQTL

^b^Minimum value

^c^Maximum value

^d^Average value

^e^Standard deviation of value

^f^Values of eQTL interval or distance shown in mega bases. Zero interval values correspond to eQTL associated with a single SNP

All autosomes had associated eQTL, with SSC9 containing the most associations (42 eQTL). We considered a single marker associated with more than ten genes to be a plausible putative hotspot, and two chromosomes contained a putative hotspot; SSC9 (ASGA0044684; SSC9:125.0-Mb) and SSC15 (H3GA0052416; SSC15:121.8-Mb). ASGA0044684 was associated with 25 transcripts, and H3GA0052416 with 11 transcripts (FDR ≤ 0.01). Both plausible hotspots mapped to non-coding regions, an intron variant of the ral guanine nucleotide dissociation stimulator like 1 (*RGL1*) gene on SSC9, and an intergenic variant on SSC15.

### Local versus distant regulators of gene expression

**Fig. 1:**
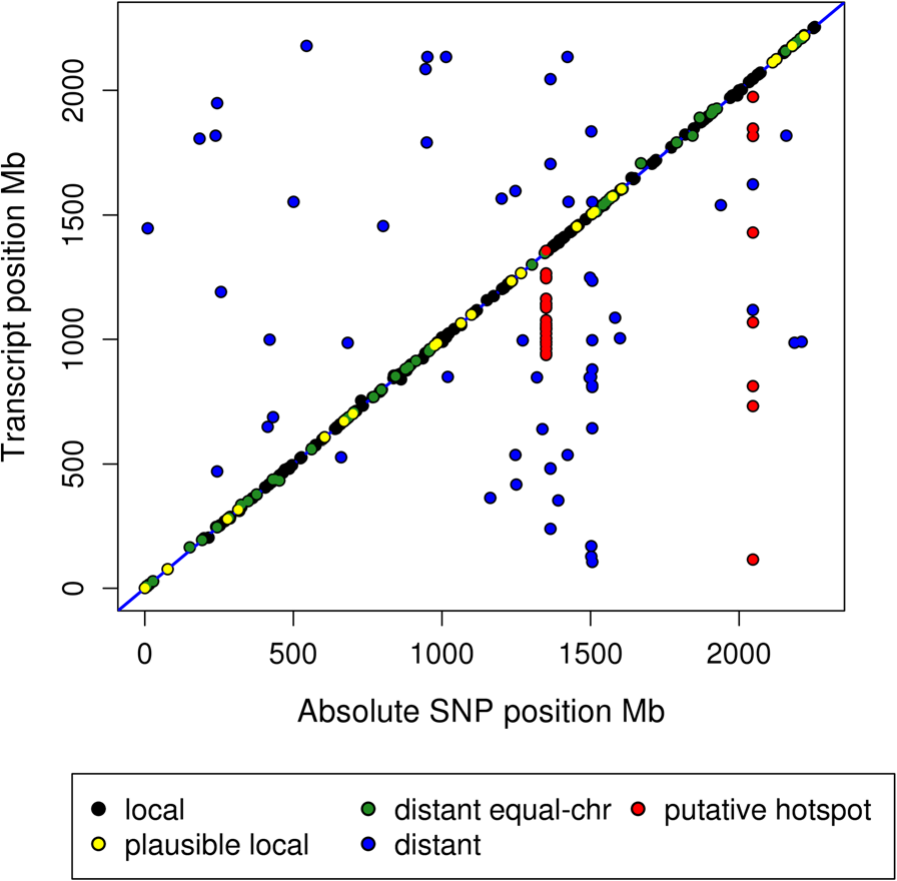
eQTL map. The y-axis represents the absolute genomic position of the gene and the x-axis represents the genomic location of its associated SNP marker. Associations aligning on the diagonal are eQTL found on the same chromosome as the gene. A plausible position range was identified for each eQTL interval based on the peak’s flanking markers, and local regulation was determined when the gene position overlapped this range, shown in black. Plausible local regulators of gene expression (described in Figure S1 in Additional file 2) are shown in yellow. The eQTL intervals shown in green are distant regulators that map to the same chromosome as their associated gene. Distant regulators mapping to a different chromosome than the associated gene are shown in blue. The eQTL shown in red are plausible putative hotspots on SSC9 and SSC15

For each of the eQTL intervals, a plausible position range delimited by the first and last significant marker (FDR ≤ 0.01) was identified and compared to the mapped position of the associated gene transcript to distinguish between local and distant regulators of gene expression (Fig. 1 and Additional file 2 Figure S1). A classification of local-acting regulator of gene expression was determined if the position of the associated gene transcript overlapped the eQTL interval (Additional file 2 Figure S1). We identified 166 local regulators of gene expression (Fig. 1, black associations).

The average distance from the gene position and peak eQTL SNP for local regulators was 1.90 ± 3.86-Mb. However, due to the large plausible position range for some local eQTL (up to 175-Mb), the maximum distance for a local regulator was 25-Mb (Table 1). If the gene mapped to the same chromosome but fell outside the range of its associated eQTL with markers below the significance threshold between the gene and eQTL positions, the eQTL was considered to be a distant regulator on the same chromosome as the associated gene (Additional file 2 Figure S1). A total of 61 distant regulators on the same chromosome as the associated gene were identified (Fig. 1, green associations) with their eQTL interval at an average distance of 9.75 ± 22.92-Mb from the associated gene position (Table 1). However, in situations where the area between the eQTL range and the associated gene transcript were found to be devoid of markers, the eQTL was considered to be a plausible local regulator (Additional file 2 Figure S1). Under this classification, 23 plausible local regulators of gene expression were identified (Fig. 1, yellow associations) with their eQTL interval at an average distance of 0.20 ± 0.40-Mb from the associated gene position (Table 1). An eQTL that mapped to a different chromosome than its associated gene transcript was classified as a distant regulator (Additional file 2 Figure S1). We observed 87 distant regulators of gene expression (Fig. 1, blue associations). A non- parametric test showed local eQTL had significantly higher numbers of associated SNPs than distant eQTL (*p*-value ≤2.20e-16).

### Heritability of gene expression

Heritability (h^2^) was estimated for all gene transcripts with 344 exhibiting significantly heritable expression (FDR ≤ 0.01, *p*-value ≤2.27e-04). The mean h^2^ for transcripts with significantly heritable expression was 0.51 ± 0.13, whereas the mean h^2^ for other transcripts was 0.09 ± 0.12 (Table 2). The relationship between the estimated h^2^ of gene expression and its significance is shown in Figure S2 in Additional file 2. Significant enrichment of genes associated with an eQTL was observed for the significantly heritable gene transcripts (*p*-value ≤2.2e-16; shown in red, Figure S2). The h^2^ of genes with an associated eQTL that were not significantly heritable was on average 0.21 ± 0.16 (shown in yellow, Figure S2 and summarized in Table 2), whereas the group of significantly heritable genes associated with an eQTL had a mean h^2^ of 0.57 ± 0.15 (Table 2). Mean heritability among the different regulator types was higher in the group of eQTL associated with local-acting regulation, 0.42 ± 0.22, and lowest in eQTL with distant-acting regulation, 0.17 ± 0.17 (Table 1). A non-parametric test showed a significant difference between heritabilities for local- and distant-acting regulators (*p*-value ≤1.08e-14).

**Table 2:** Heritability summary for all genes and genes with an associated eQTL

Significant h^2^	N	Heritability (h^2^)			
		Min	Max	Mean	SD
All Genes					
Yes^a^	344	0.184	0.968	0.508	0.133
No	14,879	2.210e-19	0.785	0.091	0.123
eQTL Genes					
Yes^a^	103	0.184	0.968	0.574	0.147
No	218	5.475e-10	0.745	0.206	0.165

^a^FDR ≤ 0.01

### Phenotypic QTL

Genomic regions significantly associated with growth [20], meat quality and carcass composition [21] traits have been previously identified in our MSUPRP. However, these analyses used an earlier assembly of the pig genome (Sscrofa10.2); therefore, we reanalyzed the 67 phenotypic traits for the F2 population (960 animals) following previous methods^21,22^ to generate an updated QTL map using the most current genome assembly (Sscrofa 11.1). Our QTL analysis of 29 growth traits identified 14 pQTL (Table S2 in Additional file 1, FDR ≤ 0.05, p-value ≤2.50e-04) for which seven were confirmed from Duarte et al. [20] and five exhibited a different peak SNP, in part because one of the SNP on SSC6 (ALGA0122657) did not have a genomic position in the new genome build. We were unable to confirm two pQTL on SSC2 for 10th rib backfat at 16-weeks and last rib backfat at 19-weeks, and one pQTL on SSC3 for birth weight that were reported in Gualdrón Duarte et al. [20]. However, we identified two new pQTL for loin muscle area at 16-weeks on SSC6 and last rib backfat at 10-weeks on SSC12. Our QTL analysis for carcass composition and meat quality traits identified 29 pQTL (Additional file 1 Table S2, FDR ≤ 0.05). Fourteen pQTL were confirmed from Casiro et al. [21] and eight exhibited a different peak SNP, in part because three SNP (SSC6: ALGA0122657, SSC11: M1GA0015491 and SSC15: MARC0047188) did not have genomic positions in the new genome build. Seven new pQTL were identified for cook yield (SSC5 and SSC8), last lumbar backfat (SSC4, SSC9 and SSC10), dressing percent (SSC11) and loin weight (SSC11). In total, 43 pQTL were mapped using the Sscrofa11.1 genome assembly, including six QTL for 10th rib backfat from 13 to 22 weeks of age, seven QTL for last rib backfat from 13 to 22 weeks of age, one QTL for loin muscle area at 16 weeks of age, 13 QTL for carcass composition traits and 16 QTL for meat quality traits.

**Fig. 2:**
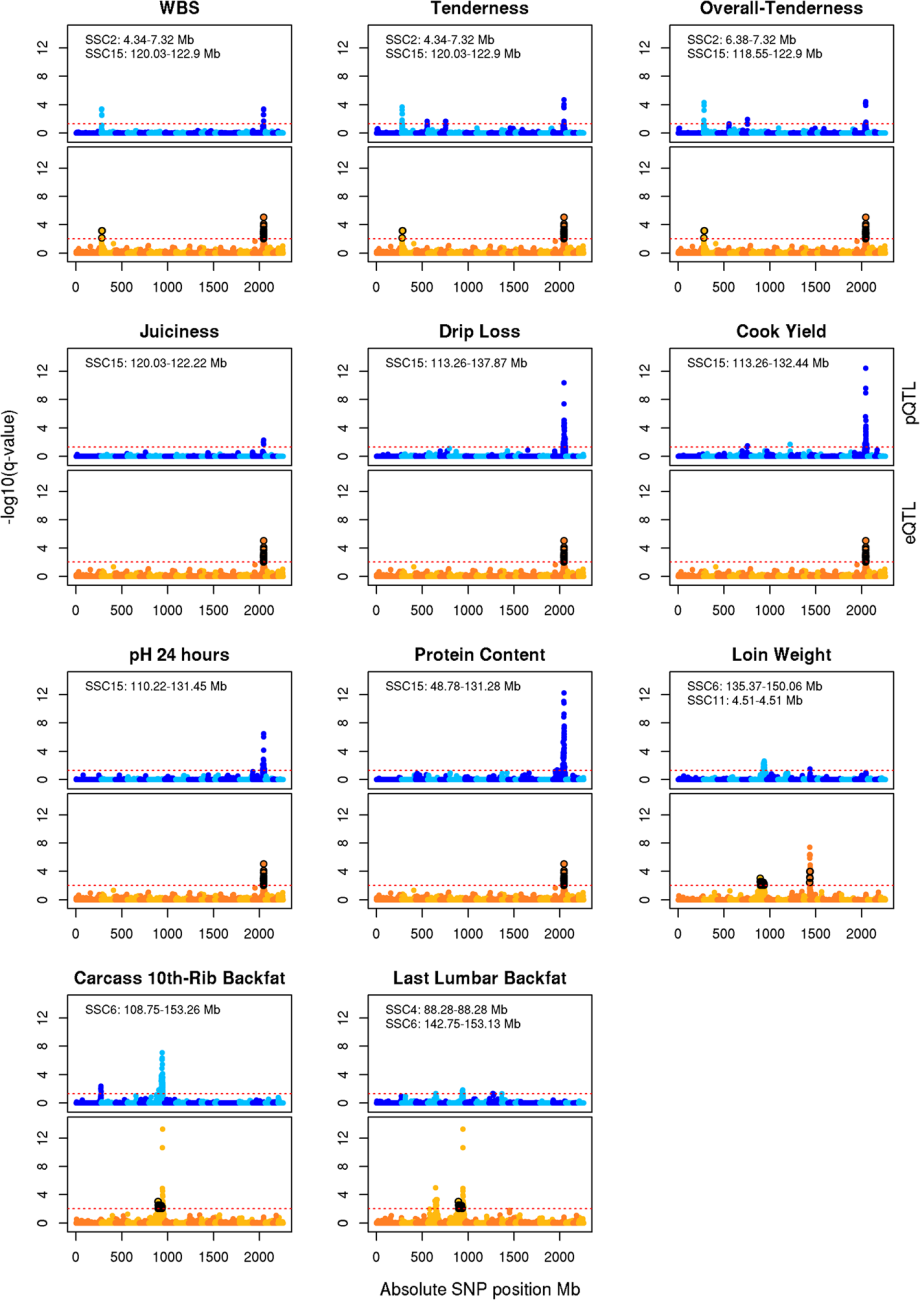
Manhattan plots of meat quality and carcass composition pQTL co-localized with eQTL. The x-axis is the absolute genome position in mega-bases. The y-axis is the negative base 10 logarithm of q-values, with the red line representing the significance threshold. Manhattan plots in shades of blue are for the pQTL (FDR ≤ 0.05), and those in shades of orange are for the eQTL (FDR ≤ 0.01). SNPs associated with an eQTL co-localizing with a pQTL, and whose association is no longer significant after performing the conditional analysis are shown in black

**Fig. 3:**
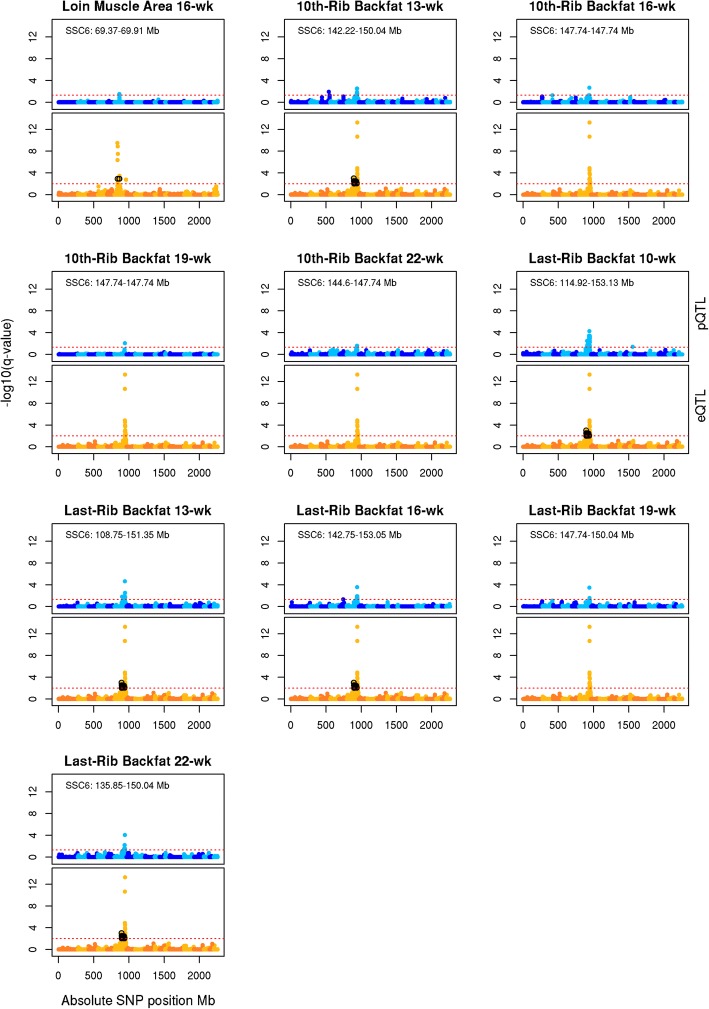
Manhattan plots of growth pQTL co-localized with eQTL. The x-axis is the absolute genome position in mega-bases. The y-axis is the negative base 10 logarithm of q-values, with the red line representing the significance threshold. Manhattan plots in shades of blue are for the pQTL (FDR ≤ 0.05), and those in shades of orange are for the eQTL (FDR ≤ 0.01). SNPs associated with an eQTL co-localizing with a pQTL, and whose association is no longer significant after performing the conditional analysis are shown in black

Meat quality traits in our population exhibited phenotypic correlations as expected. WBS was negatively correlated with sensory panel scores (i.e., juiciness, tenderness and overall-tenderness) and cook yield, and positively correlated with protein percent (*p*-value ≤8e-05, Additional file 2 Figure S3). Cook yield was negatively correlated with drip loss, and positively correlated with 24-h pH and protein percent (p-value ≤8e-05, Figure S3). Phenotypes related to tenderness were associated with QTL on SSC2, and all eight of the aforementioned correlated meat quality phenotypes were associated with QTL mapped to SSC15 (Fig. 2). A similar trend was observed for growth and carcass composition traits related to fat deposition and muscle weight where serial ultrasound measures for 10th and last rib backfat were positively correlated with carcass 10th-rib and last lumbar backfat, and negatively correlated with loin weight (*p*-value ≤8e-05, Figure S3), and these traits were associated with QTL on SSC6 (Fig. 2 and Fig. 3).

### Co-localization of phenotypic QTL with expression QTL

The association of eQTL co-localized with pQTL was performed through a conditional analysis of transcript abundance, which fixed the peak pQTL SNP, to elucidate eQTL significantly associated with phenotypic traits. Manhattan plots of eQTL co-localized with pQTL are shown in Fig. 2 for meat quality and carcass composition traits, and Fig. 3 for growth traits. The conditional analysis tested 53 eQTL (orange associations) co-localized with 34 pQTL (blue associations) for ten growth and 11 meat quality and carcass composition traits (Fig. 2 and Fig. 3; Table 3 and Additional file 1 Table S3). A total of 16 eQTL were significantly associated with 21 pQTL, where conditioning upon the peak pQTL marker resulted in the complete removal of eQTL significance (*p*-value ≤5.95e-04 for SNP effect and FDR ≤ 0.01 for eQTL significance; black associations in Fig. 2 and Fig. 3; Table 3 and Table S3 in Additional file 1). Three pQTL regions common among correlated phenotypes co-localized with eQTL, resulting in eQTL significantly associated with variation for multiple phenotypes. Pearson correlation between phenotypic values and the colocalized gene expression resulted in 11 genes with expression significantly correlated with phenotype; 73% of these genes were correlated with protein percent (Additional file 1 Table S3). The LD between peak QTL SNP for pQTL significantly colocalized with eQTL was on average 0.317 ± 0.08 (Additional file 1 Table S3).

**Table 3:** Phenotypic QTL co-localized with expression QTL

Phenotype	SSC	Peak SNP	Position	E^a^	VS^b^	h^2^	qvalue^c^	Interval^d^	N^e^
Carcass 10th-Rib Backfat	1	ALGA0010839	270.61	+	0.03	0.45	4.05e-03	270.39–274.20	1
WBS	2	M1GA0002229	4.34	–	0.04	0.26	4.07e-04	4.34–7.32	1^f^
SP Tenderness	2	H3GA0005676	6.77	+	0.05	0.29	2.02e-04	4.34–7.32	1^f^
SP Overall Tenderness	2	H3GA0005676	6.77	+	0.05	0.28	4.80e-05	6.38–7.32	1^f^
Last Lumbar Backfat	4	ASGA0092651	88.28	–	0.04	0.41	4.53e-02	0	4
Last-Rib Backfat 16-wk	5	ALGA0031990	55.01	+	0.03	0.47	4.62e-02	53.28–55.01	1
Cook Yield	5	MARC0036560	66.10	+	0.03	0.31	3.30e-02	62.76–66.10	1
Loin Muscle Area 16-wk	6	ASGA0105067	69.37	+	0.04	0.29	3.16e-02	69.37–69.91	4^f^
Last-Rib Backfat 13-wk	6	ALGA0104402	147.74	–	0.07	0.43	2.33e-05	108.75–151.35	6^f^
Carcass 10th-Rib Backfat	6	M1GA0008917	144.60	–	0.12	0.45	8.11e-08	108.75–153.26	6^f^
Last-Rib Backfat 10-wk	6	ALGA0104402	147.74	–	0.07	0.35	5.25e-05	114.92–153.13	6^f^
Loin Weight	6	ASGA0029651	144.64	–	0.06	0.30	2.24e-03	135.37–150.06	4^f^
Last-Rib Backfat 22-wk	6	ALGA0104402	147.74	–	0.07	0.49	8.80e-05	135.85–150.04	4^f^
10th-Rib Backfat 13-wk	6	ALGA0104402	147.74	–	0.05	0.43	3.05e-03	142.22–150.04	4
Last-Rib Backfat 16-wk	6	ALGA0104402	147.74	–	0.06	0.47	2.63e-04	142.75–153.05	5
Last Lumbar Backfat	6	ALGA0104402	147.74	–	0.05	0.41	1.38e-02	142.75–153.13	5
10th-Rib Backfat 10-wk	6	ASGA0029651	144.64	+	0.06	0.41	1.56e-02	144.55–147.74	2
10th-Rib Backfat 22-wk	6	ALGA0104402	147.744	–	0.04	0.47	2.69e-02	144.60–147.74	2
10th-Rib Backfat 16-wk	6	ALGA0104402	147.74	–	0.06	0.49	2.14e-03	0	2
10th-Rib Backfat 19-wk	6	ALGA0104402	147.74	–	0.05	0.50	8.72e-03	147.74–147.74	2
Last-Rib Backfat 19-wk	6	ALGA0104402	147.74	–	0.06	0.57	3.41e-04	147.74–150.04	2
Number of Ribs	7	ALGA0043983	98.51	+	0.12	0.36	4.19e-09	59.41–111.38	10
Cook Yield	8	DRGA0008986	134.93	–	0.03	0.31	2.01e-02	134.93–134.93	1
Dressing Percent	11	M1GA0014839	6.88	+	0.03	0.24	4.33e-02	1.72–6.88	2
Loin Weight	11	ALGA0060368	4.51	–	0.03	0.30	3.10e-02	4.51–4.51	2^f^
Last-Rib Backfat 10-wk	12	ASGA0054658	41.73	–	0.02	0.35	4.03e-02	41.73–41.73	2
Protein Percent	15	MARC0093624	122.22	+	0.21	0.38	8.71e-20	48.78–131.28	21^f^
24-h pH	15	MARC0093624	122.22	+	0.09	0.19	3.35e-07	110.22–131.45	16^f^
Cook Yield	15	MARC0093624	122.22	+	0.15	0.31	3.61e-13	113.26–132.44	16^f^
Drip Loss	15	MARC0093624	122.22	–	0.13	0.28	4.20e-11	113.26–137.87	17^f^
SP Overall Tenderness	15	H3GA0052416	121.81	+	0.07	0.28	3.59e-05	118.55–122.90	16^f^
WBS	15	MARC0093624	122.22	+	0.06	0.26	4.07e-04	120.03–122.90	16^f^
SP Juiciness	15	H3GA0052416	121.81	+	0.04	0.07	5.06e-03	120.03–122.22	16^f^
SP Tenderness	15	H3GA0052416	121.81	+	0.07	0.29	1.90e-05	120.03–122.90	16^f^

*SNP* significantly associated with phenotype

^a^Effect of peak pQTL SNP on phenotype, positive indicates B allele increases phenotypic trait

^b^Proportion of phenotypic variance explained by peak SNP

^c^GWAS qvalue for peak SNP

^d^Interval for pQTL: start and end position

^e^Number of eQTL co-localized with the pQTL

^f^Contains at least one eQTL significantly associated with the phenotype

**Fig. 4:**
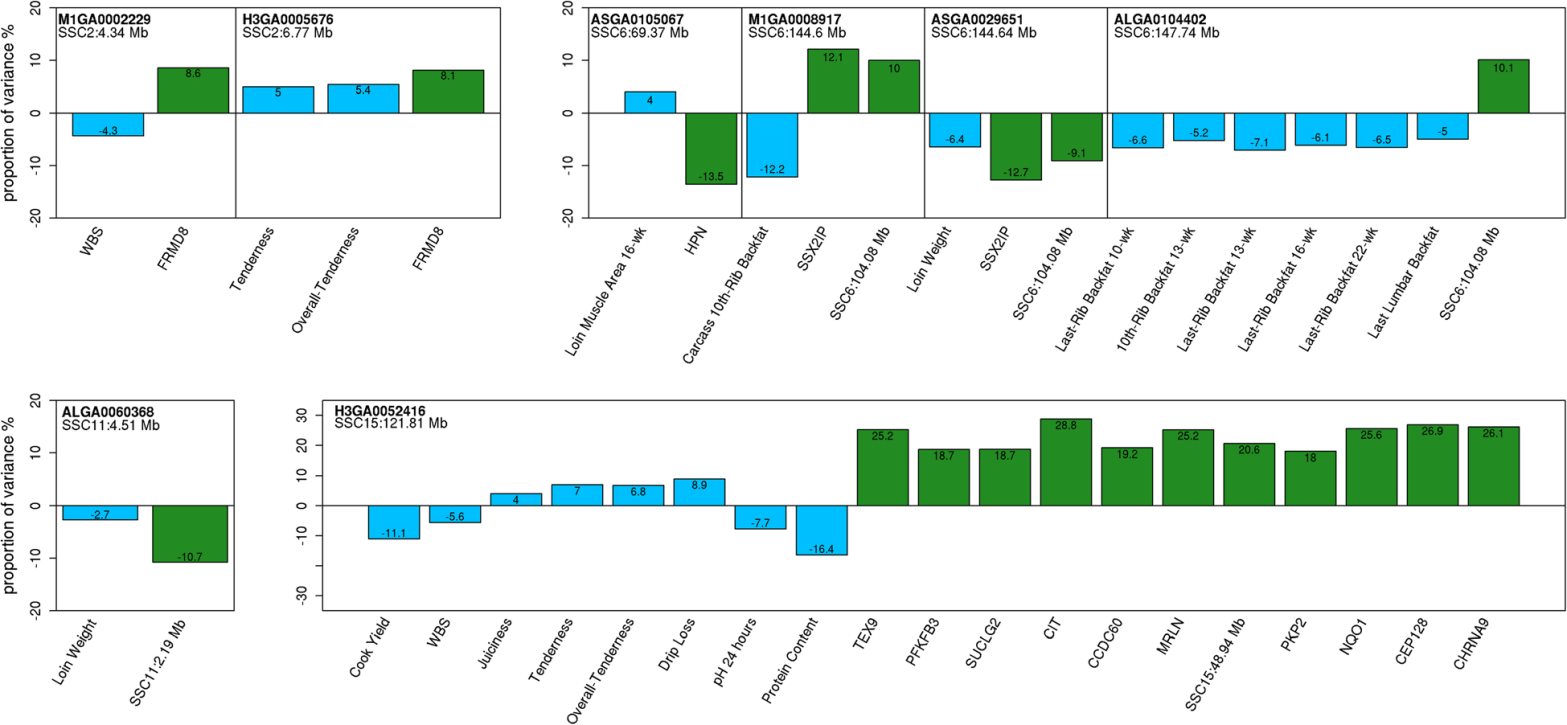
Proportion of variance explained by peak pQTL SNP for phenotypes (blue) and gene transcript abundance (green). Traits are shown on the x-axis, and the proportion of phenotypic variance explained by the SNP marker is shown on the y-axis. Directionality of bar plots indicates SNP effect on phenotype or gene expression (i.e., increase or decrease)

Phenotypic QTL for growth and carcass composition traits associated with eQTL on SSC6 revealed two genomic regions. A 28.82-Mb region (SSC6:43.819–72.625-Mb) was associated with the hepsin gene (*HSN*) and with loin muscle area at 16 weeks. A 53.33 Mb region (SSC6:99.932–153.261-Mb) was associated with a novel transcript (SSC6:104.08) and with serial ultrasound measures of last rib backfat (at 10, 13, 16 and 22 weeks of age), 10th rib backfat at 13 weeks of age, and carcass last lumbar backfat. The peak pQTL marker for loin muscle area at 16 weeks of age, ASGA0105067, accounted for 4% of the phenotypic variance and 13.5% of the gene expression variance with increased loin muscle area associated with decreased expression of the *HPN* gene (Fig. 4). The pQTL marker for backfat deposition, ALGA0104402, accounted for 5–7.1% of the phenotypic variance, and 10.1% of the gene expression variance, with increased expression of the novel transcript SSC6:104.08 associated with reduced backfat deposition (Fig. 4).

Two additional pQTL for carcass composition phenotypes (carcass 10th rib backfat and loin weight) also mapped to the 53.33-Mb region on SSC6 and were significantly associated with the SSC6:104.08 novel transcript and with SSX2IP. The peak pQTL marker for carcass 10th rib backfat (M1GA0008917) accounted for 12.2% of the phenotypic variance, with increased expression of both the SSC6:104.08 novel transcript and *SSX2IP* associated with reduced 10th rib backfat. For loin weight, the peak pQTL marker (ASGA0029651) was associated with reduced loin weight and reduced expression of both the SSC6:104.08 novel transcript and *SSX2IP,* accounting for 6.4% of the phenotypic variance and up to 12.7% of the transcript expression variance (Fig. 4). A second pQTL for loin weight was mapped on SSC11 and was significantly associated with a novel transcript (SSC11:2.19), which coincides with the uncharacterized locus LOC110255792. The peak pQTL marker for loin weight on SSC11 (ALGA0060368) accounted for 2.7% of the phenotypic variance and 10.7% of the gene expression variance. Reduced loin weight was associated with reduced expression of the SSC11:2.19 transcript (Fig. 4).

Considering the pQTL for meat quality and carcass composition traits with their associated eQTL reveals two genomic regions of particular note. A 7.90-Mb region on SSC2:4.341–12.242-Mb was associated with the FERM domain-containing 8 gene (*FRMD8*) and WBS, sensory panel tenderness and overall tenderness phenotypes, and a 110.21 Mb region on SSC15:27.666–137.874-Mb was associated with 11 genes and eight meat quality or carcass composition phenotypes (Tables 3 and 4). Significant negative correlations were observed between WBS and all three sensory panel phenotypes as expected for these traits (*r* = − 0.44 ± 0.14, *p*-value ≤8e-05, Fig. 4); more force needed to break myofibers (i.e., higher shear force values) was correlated with lower meat tenderness based on subjective scores evaluated by a trained sensory panel. The peak pQTL markers, M1GA0002229 and H3GA0005676, for meat quality traits on SSC2 accounted for approximately 5 % of the phenotypic variance and 8 % of *FRMD8* gene expression variance (Fig. 4) with increased expression of *FRMD8* associated with increased sensory panel tenderness and overall tenderness scores and decreased WBS. High linkage disequilibrium (LD) was observed between these two SNPs (*r* = 0.64).

**Table 4:** Expression QTL significantly associated with phenotypic traits

Gene	SSC Gene	h^2^	SSC eQTL	Interval eQTL	Regulator^b^	^Peak eQTL^ SNP	Position	E^c^	value^d^	Phenotype^e^
TEX9	1	3.76e-01	15	27.666–122.219	Distant	H 3GA 0052416	121.806	+	6.09e-04	M eat Quality, Protein Percent
FRM D8	2	4.00e01	2	6.383–12.242	Local	ASGA0098756	12.242	–	7.25e-04	WBS, SP Tenderness, SP Overall Tenderness
PK P2	5	2.91e-01	15	121.806–121.873	Distant	H 3GA 0052416	121.806	–	9.47e-03	M eat Quality, Protein Percent
NQO1	6	3.28e-01	15	121.806–122.219	Distant	H 3GA 0052416	121.806	+	1.74e-04	M eat Quality, Protein Percent
H PN	6	3.62e-01	6	43.809–72.625	Local	ALGA0113420	68.085	+	1.20e-03	Loin MuscleArea16-wk Last-Rib Backf at 10, 13, 22
M ETTL4	6	1.70e-01	6	99.932–136.022	Local	MA RC0107785	101.29	–	9.55e-04	wk, Carcass 10th- Ri b Backf at, Loin Weight
SSX 21 P	6	4.60e-01	6	120.497–143.338	Local	ALGA0104761	141.317	+	5.27e-03	Carcass 10th- Rib Backf at, Loin Weight
CEP128	7	4.47e-01	15	121.562–122.219	Distant	H 3GA 0052416	121.806	+	1.39e-04	M eat Quality, Protein Percent
CH RNA 9	8	4.21 e-01	15	120.031–122.219	Distant	D I A S0000678	121.562	–	7.96e-05	M eat Quality, Protein Percent
PFKFB3	10	7.44e-03	15	121.806–121.873	Distant	M A RC0027291	121.873	–	5.03e-03	M eat Quality, Protein Percent
SSC11:2.19^a^	11	5.23e-01	11	0.215–5.406	Local	ALGA0060277	2.798		1.05e-04	Loin Weight
SUCLG2	13	2.34e-01	15	120.031–121.873	Distant	M A RC0027291	121.873	–	1.06e-03	M eat Quality, Protein Percent
CI T	14	1.90e-01	15	121.562–122.219	Distant	H 3GA 0052416	121.806	+	9.14e-06	M eat Quality, Protein Percent
CCDC60	14	3.90e-01	15	121.806–122.767	Distant	H 3GA 0052416	121.806	+	1.67e-03	M eat Quality, Protein Percent
M RLN	14	4.00e-01	15	121.562–122.219	Distant	M A RC0027291	121.873	–	1.54e-04	M eat Quality, Protein Percent
SSC15:48.94^a^	15	1.75e-01	15	121.562–121.873	Distant SC	H 3GA 0052416	121.806	+	4.67e-03	M eat Quality, Protein Percent

^a^Novel gene transcripts: *Sus scrofa* chromosome and start position

^b^Regulator type for eQTL

^c^Effect the peak eQTL marker has on the gene’s expression: positive indicates B allele increases and negative indicates B allele decreases expression

^d^qvalue of peak eQTL marker (FDR < 0.01)

^e^Phenotypes significantly associated with the gene’s expression. Meat Quality includes the phenotypes for sensory panel juiciness, tenderness and overall tenderness, Warner Bratzler Shear Force, Cook Yield, Drip Loss and 24-h pH

Eleven of the eQTL significantly associated with pQTL were distant regulators of gene expression, and all of these were also associated with a plausible hotspot within the 110.21-Mb region on SSC15. The SSC15 plausible hotspot marker H3GA0052416 was the peak pQTL marker for sensory panel juiciness, tenderness and overall tenderness (Table 3), as well as the peak eQTL marker for seven gene transcripts (Table 4). The peak pQTL marker for WBS, 24-h pH, cook yield, drip loss and protein percent on SSC15 (MARC0093624) is in high LD with the H3GA0052416 marker (Pearson correlation 0.89). The expression of eight (*CEP128*, *CHRNA9*, *MRLN, NQO1*, *PFKFB3*, *PKP2*, *SUCLG2*, *TEX9*) of the eleven genes associated with the H3GA0052416 marker were significantly correlated with the protein percent phenotype (*r* = − 0.34 ± 0.05, FDR ≤ 0.05; Table S4 in Additional file 1). These results suggest a potential candidate variant(s) on SSC15 accounting for a significant portion of phenotypic variation for meat quality and carcass composition phenotypes, as well as individual gene expression variation. Since the two markers (H3GA0052416 and MARC0093624) are in high LD, the proportion of phenotypic and gene expression variance was estimated for the H3GA0052416 marker for all eight phenotypes and eleven gene transcripts including *CCDC60, CEP128*, *CHRNA9, CIT*, *MRLN, NQO1*, *PFKFB3*, *PKP2*, *SUCLG2*, *TEX9,* and a novel transcript SSC15:48.94-Mb (mapped to the uncharacterized locus LOC110257028). The H3GA0052416 marker accounted for 4–16% of the phenotypic variance and approximately 23% of the gene expression variance (Fig. 4). The B allele of the H3GA0052416 marker was associated with increased expression of the eleven genes, and was also associated with an increase in sensory panel scores and drip loss, and a decrease in WBS, 24-h pH, cook yield and protein percent (Fig. 4).

The gene protein kinase AMP-activated non-catalytic subunit gamma 3 (PRKAG3) maps to this region of SSC15, and variants of PRKAG3 have been implicated in affecting meat quality phenotypes [22, 23]. We genotyped all F2 animals for two PRKAG3 coding SNPs [21] and included these SNP in our GWAS. However, the eQTL scan did not reveal associations with either of the PRKAG3 markers. To further assess the effect of PRKAG3, we performed a conditional analysis to estimate the significance of these markers on identified eQTL (Additional file 1 Table S5). Only one gene, NQO1, was significantly associated with the PRKAG3 T30 N SNP (FDR ≤ 0.01), where T30 N accounted for up to 12% of the gene expression variance (data not shown). Given the high signal of the H3GA0052416 marker on SSC15 for various genes and meat quality traits, we estimated the proportion of phenotypic variance explained by both H3GA0052416 and the PRKAG3 T30 N marker for meat quality and carcass composition traits (Additional file 2 Figure S4). The PRKAG3 T30 N marker accounted for 0.1–2% of phenotypic variance for meat quality traits, whereas the H3GA0052416 marker accounted for 2–14%. This analysis shows the H3GA0052416 marker accounts for a greater proportion of phenotypic variance than the PRKAG3 T30 N SNP.

### RT-qPCR confirmation of CHRNA9

The statistical model identified 24 eQTL mapped to a 125-Mb region on SSC15. Eleven of these eQTL co-localized with pQTL for meat quality and carcass composition traits, and among these the *CHRNA9* gene was selected for verification using RT-qPCR (Fig. 4). *CHRNA9* is implicated in catecholamine secretion and the adaptive response to chronic stress [24], and is essential for muscle contraction [25]. The genomic position of the *CHRNA9* gene is on SSC8: 31.44–31.51-Mb, and the eQTL associated with this gene mapped to SSC15, therefore exhibiting distant-acting regulation of *CHRNA9* gene expression. RT-qPCR was performed to confirm the expression pattern of the *CHRNA9* gene in *longissimus dorsi* muscle. Pearson correlation between the ∆Ct and RNA-seq log-cpm for *CHRNA9* transcript abundance was − 0.58. The marker DIAS0000678 was significantly associated with both RNA-seq and ∆Ct for *CHRNA9* (*p*-value ≤4.23e-06), exhibiting a significant dominant effect with the B allele associated with increased *CHRNA9* transcript abundance (*p*-value ≤0.05, Additional file 1 Table S6).

## Discussion

For this study, we identified eQTL for *longissimus dorsi* muscle transcripts from pigs in an F2 resource population, and we declared local versus distant eQTL effects based on LD structure. When an eQTL and the associated gene are located on the same chromosome, the low resolution of the swine genome due to long range LD [26, 27] limits the ability to distinguish between cis-acting and trans-acting eQTL. Most eQTL association studies use a fixed distance threshold between the position of the eQTL interval and the gene transcript to define cis-acting (i.e., local) versus trans-acting (i.e., distant) regulation. For instance, distance thresholds between 1-Mb and 10-Mb have been used in recent pig eQTL maps [10, 13, 28–31]. Human eQTL scans have used more conservative distance thresholds of 100-Kb – 500-Kb between gene position and eQTL to declare local regulation [32, 33]. A shorter local threshold is logical for human eQTL studies because they typically show higher resolution due to increased SNP density (millions of genotyped markers [32]), and the extent of LD is less than in livestock populations due to greater genetic diversity in human populations [33]. In this study, we present an alternative to the use of a fixed distance for declaring local versus distant eQTL effects. This is important because the range of a mapped eQTL will depend on the LD pattern at the QTL genomic position. Building upon previous approaches to determine local regulation [11, 15, 34, 35] in eQTL linkage maps, this study considered the significance of each individual marker surrounding the plausible position range of the eQTL interval to distinguish between local and distant modes of action. In cases where there are no genotyped markers between the plausible position of the eQTL interval and the location of the associated gene, there is not sufficient information to determine local versus distant; here we consider this scenario as plausible local regulation. We note that in our study the median distance between plausible local eQTL regulators and their associated gene was 31-Kb, which is a shorter distance than eQTL designated as local for other pig eQTL mapping studies [10, 13, 28–31]. Therefore, it is feasible that most of these regulators may be acting locally, since cis-acting transcription factor binding sites have been found located ~ 100 kb from the mapped position of a gene transcript [36]. However, without a denser SNP set and/or a larger population size, we cannot definitively identify the mode of action of these eQTL. A potential way to further investigate if these eQTL are acting locally or distantly would be through allele-specific expression analyses [14].

Heritability of gene expression contributes to our understanding of the inheritance of gene expression regulation. Estimating the heritability of gene expression is common in human eQTL studies to elucidate the genetic contribution of gene expression variation and its influence on the divergence of complex traits [32, 33, 37, 38]. Human studies have shown higher heritability estimates for housekeeping genes and genes with local eQTL, whereas genes with distant eQTL tend to exhibit lower heritability [32, 37, 38]. Bryois et al. [37] suggested a fraction of missing heritability may be due to common variants with both local and distant effects on gene expression, with the latter being of small effect size. Examples of local eQTL with large distant effects in human studies include variants influencing the expression of transcription factor genes or histone methyltransferase genes [37]. Heritability of gene expression has not been emphasized in pig eQTL studies, except for one report where heritability was used as a filtering criteria to prioritize genes [35]. In this study, we estimated narrow-sense heritability for all gene expression profiles and determined significance with likelihood ratio tests. Consistent with previous studies in humans, the observed heritabilities for genes with distant eQTL were significantly lower than for locally regulated genes [32]. This trend is consistent with previous findings where genes influenced by many distant factors of small effect tend to exhibit lower heritability than genes with local regulation. Testing for significant additive genetic effects of transcript abundance in outbred animal populations requires a large sample size to increase power to detect smaller effects. In our GWA scan, we were able to capture the variance associated with gene transcripts subject to genetic control with low heritability. A previous eQTL scan performed with 57 muscle tissue samples from an F2 swine population observed an average heritability of 0.45 for eQTL genes [35]. While this value is greater than the average heritability observed in our study (0.32), Liaubet et al. [35] limited the eQTL scan to gene transcripts with heritability greater than 0.05. The use of a heritability threshold to filter genes in eQTL studies may miss potential associations, especially those of low effect such as distant eQTL, which we show to have lower average heritability estimates.

We identified three gene transcripts that were associated with pQTL for fat deposition and carcass composition traits on SSC6. One of these eQTL genes, synovial sarcoma X breakpoint 2 interacting protein (*SSX2IP*), was significantly associated with pQTL for carcass 10th rib backfat and loin weight. An eQTL was previously identified for this gene on SSC6 using microarray data from the same animals used in this study, and consistent with our results, Peñagaricano et al. [39] reported a negative causal effect of increased expression of *SSX2IP* on backfat thickness [39]. In addition, *SSX2IP* has been associated with waist to hip ratio, a common measure of body fat distribution, in women of African descent [40].

The genes associated with pQTL for tenderness phenotypes on SSC2 or meat quality phenotypes on SSC15 share biological processes known to directly influence the organoleptic properties of meat, including calcium signaling (*FRMD8*, *MRLN*, *PKP2* and *CHRNA9*), energy metabolism (*SUCLG2* and *PFKFB3*), redox hemostasis (*NQO1* and *CEP128*) and cytoskeletal structure (*CIT* and *CCDC60*). One of the genes related to calcium signaling is the FERM domain containing 8 (*FRMD8*) gene associated with pQTL for WBS, and sensory panel tenderness and overall tenderness on SSC2. Two independent GWAS, one in a crossbred commercial pig population [41] and another in a multigenerational Landrace-Duroc-Yorkshire composite population [42], reported QTL for slice shear force (a technique similar to WBS) in the same genomic region as this study. Zhang et al., [41] identified *FRMD8* to be one of four genes in the region to play a role in pork tenderization, and the peak SNP reported by Nonneman et al. [42] was the same peak SNP identified in our analysis (H3GA0005672). We showed with our conditional analysis that increased expression of *FRMD8* was associated with improvements in pork tenderness. *FRMD8* is a member of the FERM (Four-point-one, Ezrin, Radixin, Meosin) protein superfamily known to possess both structural and signaling functions including numerous protein-binding interactions mainly in the cytoskeleton of cells [43]. This includes interactions with transmembrane ion channels and membrane lipids including the phosphatidylinositol 4,5-bisphosphate (PIP_2_). PIP_2_ is the precursor of inositol 1,4,5-triphosphate (IP_3_) involved in Ca^2+^ signaling [44–46] and IP_3_ has been suggested as a potential indicator of meat tenderness in beef cattle [47]. The activation of the PIP_2_ Ca^2+^ signaling system controls diverse cellular processes in numerous tissues [48]. In skeletal muscle the sarcoplasmic reticulum ryanodine receptor is the Ca^2+^ release channel, however, PIP_2_ has been localized to the transverse tubular membrane, and IP_3_ receptors have been found in differentiated muscle fibers and implicated in excitation-contraction coupling (for review see Csernoch et al. [49]). Thus, *FRMD8* may play a role in Ca^2+^ signaling and excitation-contraction coupling of skeletal muscles through interactions with PIP_2_.

Similar to *FRMD8,* the *MRLN* gene is also implicated in muscle contraction. *MRLN* encodes myoregulin, a micropeptide inhibitor of the sarco/endoplasmic reticulum Ca^+ 2^ ATPase (SERCA). SERCA regulates relaxation after muscle contraction, specifically by pumping Ca^+ 2^ back to the sarcoplasmic reticulum. Binding myoregulin to SERCA lowers its affinity to Ca^+ 2^, reducing the rate of Ca^+ 2^ reuptake into the sarcoplasmic reticulum [50]. Increased expression of *MRLN* was associated with improvements in pork tenderization, decreased 24-h pH and increased drip loss in our study. The observed effect of *MRLN* gene expression on meat quality phenotypes may be due to its involvement in regulating muscle contractility and calcium signaling, which have a direct effect on postmortem proteolysis.

Additional genes implicated in calcium signaling and associated with meat quality phenotypes and the H3GA0052416 marker were the *PKP2* and *CHRNA9* genes. *PKP2* encodes a plakophilin protein known to localize to cell desmosomes and nuclei, and play a role in linking cadherins to intermediate filaments in the cytoskeleton. In mouse cardiac muscle, *PKP2* has been shown to regulate the transcription of genes controlling intercellular calcium homeostasis, and reduced expression of *PKP2* decreases the expression of several calcium signaling genes including the cardiac muscle ryanodine receptor [51]. In this study, increased expression of *PKP2* was associated with improvements in pork tenderness, and decreases in 24-h pH, protein percent and cook yield, suggesting a role for this gene in modulating skeletal muscle calcium signaling during the conversion of muscle to meat. The *CHRNA9* gene is one of sixteen subunits of the nicotinic acetylcholine receptor (AChR). These ligand-gated ion channels permit the transmission of presynaptic acetylcholine release and postsynaptic excitatory potential. Found only in neuronal tissue, *CHRNA9* is one of three AChR containing only α subunits [25] (α9-AChR), and in neuromuscular junctions AChR are essential for muscle contraction [25]. Since α9-AChR possess higher calcium permeability, they play a role in catecholamine secretion and the adaptive response to chronic stress [24]. In this study, increased expression of *CHRNA9* was associated with improved tenderness scores, increased drip loss, and decreased cook yield, protein percent and 24-h pH. In addition, we verified the expression of *CHRNA9* in skeletal muscle with RT-qPCR and confirmed a significant dominance effect of the peak eQTL SNP (DIAS0000678) on *CHRNA9* gene expression. Changes in the expression of *CHRNA9* may potentially regulate the postsynaptic excitatory potential during the conversion of muscle to meat thereby influencing Ca^2+^ release to the cytoplasm, apoptotic mitochondrial changes and proteolytic enzymatic activity.

Additional genes associated with meat quality traits on SSC15 (*PFKFB3*, *CEP128*, *NQO1* and *SUCLG2*) were implicated in biological processes related to redox homeostasis and energy metabolism. The *PFKFB3* gene regulates the synthesis and degradation of fructose-2, 6-bisphosphate and fructose-6-phosphate in the process of glucose metabolism. The promoter of the *PRKFB3* gene contains hypoxia-inducible factor-1 (HIF-1) binding sites [52]. The transcription factor HIF-1 is a master regulator of oxygen homeostasis by activating several downstream pathways including the mitogen-activated protein kinase (MAPK), mammalian target of rapamycin (mTOR), phosphoinositide 3-kinase-protein kinase B (PI3K-Akt), vascular endothelial growth factor (VEGF) and calcium signaling pathways, as well as anaerobic metabolism. *PFKFB3* is consistently overexpressed in many tumor cells, and knockdown of *PFKFB3* promotes apoptosis of tumor cells [52]. Rapidly proliferating tumor cells have the ability to increase glucose uptake by using anaerobic glycolysis as the primary source of energy, known as the Warburg effect. Taken together, *PFKFB3* is critical for cell proliferation and survival by regulating glucose metabolism and prevents apoptosis through the activation of cyclin-dependent kinases [52, 53]. No reports have suggested a role for PRKFB3 in meat quality. However, in our study, increased expression of *PRKFB3* was associated with increased pork tenderness. Thus, similarly to PRKAG3, PRKFB3 may be involved in postmortem glycolytic potential.

The CEP128 gene is related to the PI3K-Akt-mTOR signaling pathway. Centrosomal protein 128 (*CEP128*) is part of the centrosomal protein family, including *CEP55* that has been implicated in cancer progression [54]. Mutations within *CEP128* have been associated with an aggressive type of lymphoma, the diffuse large B-cell lymphoma (DLBCL) [55]. Functional gene studies have not been performed for *CEP128*, however mutations identified in refractory DLBCL patients, including those in *CEP128*, were associated with PI3K-Akt-mTOR signaling pathways and increased mitochondrial oxidative phosphorylation, and play an important role in treatment resistance [55]. The PI3K-Akt-mTOR pathway is upregulated in cancer cells, controlling the survival and proliferation of these cells. In our study, increased expression of *CEP128* was associated with improved tenderness scores, potentially involving PI3K/Akt/mTOR signaling.

The Edomucin (*EMCN*) gene associated with a local-acting eQTL on SSC8 plays a critical role in angiogenesis. Angiogenesis is the process of new blood vessel formation with its key regulator, vascular endothelial growth factor (VEGF), triggering downstream signaling cascades including MAPK-ERK1/2, PI3k/Akt and p38-MAPK pathways [56]. These signaling pathways promote endothelial cell migration, proliferation, and survival and are activated by HIF-1 which induces VEGF expression [57]. While this eQTL is not directly associated with a phenotype in our population, it is connected to the pathways regulated by the genes associated with the H3GA0052416 marker on SSC15.

The remaining two genes, *NQO1* and *SUCLG2*, were associated with improvements in meat tenderization and pH decline. The nuclear erythroid-2-p45-related factor-2 (Nrf2) is a transcription factor known to regulate redox homeostasis and anti-inflammatory response by controlling the expression of Phase I and Phase II anti-oxidant enzymes containing the antioxidant response element (ARE; cis-acting regulatory or enhancer sequence) in their promoter regions. *NQO1* (NADPH quinone oxidoreductase-1) is one of these enzymes whose expression is induced by Nrf2 in several tissues [58–61]. Consequently, knockdown of Nrf2 has been reported to significantly decrease expression of *NQO1* in both mouse skeletal muscle [60] and C2C12 mouse myotubes [61]. In early postmortem muscle, the antioxidant defense system is speculated to influence proteolysis and thereby meat tenderization [5]. Increased expression of *NQO1* in this study was associated with several meat quality traits including tenderness, pH and drip loss phenotypes implying a significant role in post-mortem proteolysis. The succinate-CoA ligase GDP-forming beta subunit (*SUCLG2*) has been implicated in the *SUCLG1*-related mitochondrial DNA depletion syndrome affecting brain and skeletal muscle tissues. Individuals affected by this syndrome present an array of symptoms including spasmodic muscle contractions, contracture or destruction of muscle cells and hypoglycemia [62]. Knockdown of the *SUCLG2* gene in fibroblasts was reported to decrease mitochondrial DNA, mitochondrial nucleoside diphosphate kinase and cytochrome c oxidase activities [63]. These results highlight the critical role *SUCLG2* plays in mitochondrial DNA maintenance and ATP production. In our study, increased expression of *SUCLG2* was associated with improvements in meat quality traits suggesting a potential role in regulating ATP production and postmortem pH decline.

In addition to genes involved in specific biological functions, genes encoding structural proteins were also observed to be associated with the H3GA0052416 marker on SSC15 (*CIT* and *CCDC60*). *CIT*, citron Rho-interacting serine/threonine kinase, is considered to be a scaffold protein that binds to several mitotic proteins, and knockout of *CIT* leads to cytokinetic defects. One such protein-protein interaction involves the two-pore channel 1 (*TPC1*), which Horton et al. [64] reported to cause disruption in myosin light chain phosphorylation (pMLC). In skeletal muscle, pMLC has been associated with age-related muscle dysfunction [65], and decreased pMLC is associated with a reduced fraction of myosin heads interacting with thin filaments [65]. Thus, increased expression of *CIT* could potentially increase muscle breakdown, which is consistent with our findings where higher expression of *CIT* was associated with improvements in pork tenderization, and reduced protein content and cook yield. CCDC60 is a coil-coil domain protein, which are believed to act as “cellular velcro” holding together molecules, cellular structures and tissues [66]. The biological function of CCDC60 is unknown, but recent GWAS have associated this gene with the neurological disorder schizophrenia in humans [67]. Proteomic analysis of post-mortem pre-frontal cortex of schizophrenia patients and non-schizophrenia individuals identified differentially expressed proteins involved in calcium homeostasis, cytoskeleton assembly and energy metabolism [68]. Therefore, it is feasible that similar functions may occur in skeletal muscle tissue. In this study, increased expression of *CCDC60* was associated with tenderness, pH, cook yield and drip loss phenotypes implicating the role of this gene in the conversion of muscle to meat.

Eleven eQTL genes were enriched in pQTL for meat quality traits on SSC15 (*PFKFB3*, *SUCLG2*, *CIT*, *CCDC60*, *MRLN*, *PKP2*, *NQO1*, *CEP128*, *CHRNA9*, *TEX9* and a novel transcript SSC15:48.94). The novel transcript mapped to an uncharacterized locus, LOC110257028, on SSC15. The other ten gene transcripts mapped to different chromosomes than their associated eQTL. These results illustrate the advantage of the joint analysis of gene expression profiles and trait phenotypes to uncover the genetic architecture of polygenic traits. In this study, increased expression of the 11 genes was associated with improvements in meat quality phenotypes. Moreover, this QTL region harbors a plausible putative hotspot (H3GA0052416) regulating the expression of all 11 gene transcripts. Breitling et al. reported the high false positive rate associated with hotspot discovery. Therefore, to mitigate this, we used a higher threshold of significance to detect eQTL. The H3GA0052416 marker on SSC15 was also associated with multiple meat quality phenotypes. The high correlation observed between the 11 gene expressions, and between the eight meat quality phenotypes suggests the potential that these associations are due to a master regulator on SSC15 (i.e., a putative hotspot). The *PRKAG3* gene has been suggested as such a regulator of meat quality traits in pigs. PRKAG3 regulates glycogen potential, which has a cascading effect in postmortem metabolism. The SNP panel used in this study does not have sufficient coverage of the PRKAG3 gene. To address this, our F2 population was genotyped for two known PRKAG3 SNPs [21]. However, *PRKAG3* did not explain the relationship observed in the putative hotpot. A missense polymorphism within the *PRKAG3* gene, T30 N SSC15:120.865-Mb, was significantly associated with just one of the 11 genes, *NQO1*, despite showing significant association with all eight meat quality phenotypes in this population [21].

## Conclusions

In summary, the joint analysis of pQTL with eQTL from our well-characterized pig resource population identified molecular markers significantly associated with both economically important phenotypes and gene transcript abundance. This approach revealed both local- and distant-acting regulators of gene expression influencing meat quality, carcass composition and growth traits. These phenotypic traits are correlated, and we show how correlated phenotypes exhibit correlated gene expression measured through a plausible hotspot contained within QTL regions for both expression and phenotypic traits. We highlight novel candidate genes with specific roles in cytoskeletal structure and signaling pathways regulating meat quality phenotypes including redox hemostasis (*NQO1* and *CEP128*), energy metabolism (*SUCLG2* and *PRKFB3*), Ca^2+^ signaling (*FRMD8, MRLN, PKP2* and *CHRNA9*) and cytoskeletal structure (*CIT* and *CCDC60*) during the initial conversion of muscle to meat. Taken together the identified genes and their associated functions and pathways increase our knowledge of the genomic architecture of meat quality phenotypes.

## Methods

### Pig population and phenotype collection

Animal housing and care protocols were evaluated and approved by the Michigan State University All University Committee on Animal Use and Care (AUF # 09/03–114-00). The experimental design, phenotyping and sample collection for the MSUPRP has been reported previously [17–19]. All MSUPRP pigs were reared at the Michigan State University Swine Teaching and Research Center, and pigs for this study were euthanized by humane slaughter in a USDA inspected abattoir at Michigan State University. The MSUPRP was developed from 4 Duroc boars and 15 Pietrain sows [18, 19]. From the F1 progeny, 56 animals (6 males and 50 females) were retained to produce the F2 generation, which included 1259 animals from 142 litters. A total of 67 phenotypic traits were collected for the F2 generation [18, 19]. A subset of the F2 pigs (168) were selected for this study using a selective profiling scheme based on extremes in loin muscle area and backfat thickness phenotypes within litter (44 litters) and sex [69]. Summary statistics for the 67 phenotypic traits (29 growth traits, 20 carcass composition traits and 18 meat quality traits) in the F2 population, and the subset of animals used for this study are shown in Additional file 1 Table S7.

### Genotyping

SNP genotypes for the MSUPRP were available from prior studies [70, 71]. Genotyping was performed by Neogen Corporation - GeneSeek Operations (Lincoln NE) using the Illumina PorcineSNP60 BeadChip [72] for the F0, F1 and ~ 1/3 of the F2 population (including all F2 pigs used for the eQTL analysis), and the GeneSeek Genomic Profiler for Porcine Low Density (GGP-Porcine LD) for the remaining F2 pigs [70, 71]. Missing genotypes were imputed with an accuracy of 0.97 [70, 71]. Monomorphic markers and non-autosomal markers were eliminated from further analysis, as were those showing divergence from Mendelian inheritance rules. An updated genomic map for SNPs on the Sscrofa11.1 genome assembly was obtained from Neogen (Lincoln NE). Additional filtering was performed to exclude markers with a minor allele frequency lower than 0.01 and to reduce the degree of correlation between adjacent markers (i.e., if a pair of neighboring markers had a correlation of allelic dosage greater than 0.95, one of the two markers was eliminated; this filtering was performed only for the eQTL analysis). Filtering resulted in 23,162 markers for the eQTL analysis and 43,130 markers for the pQTL analysis. Two coding SNPs in the protein kinase AMP-activated non-catalytic subunit gamma 3 (*PRKAG3*) gene, I199V and T30 N [22, 23], were also genotyped in the MSUPRP as previously described in Casiro et al. [21].

### RNA extraction and RNA sequencing

Tissue samples were collected immediately post mortem from the *longissimus dorsi* muscle, flash frozen in liquid nitrogen and stored at − 80 °C until processed [17]. RNA extraction was performed with the miRNeasy Mini Kit (Qiagen, Germantown, MD) following the manufacturer’s protocol. Quality and quantity of extracted total RNA were determined using the Agilent 2100 Bioanalyzer (RIN ≥ 7). Sequencing was performed at the Michigan State University Research Technology Support Facility. Libraries for 24 samples were prepared using the Illumina TruSeq RNA Library Prep Kit v2, and sequenced on the Illumina HiSeq 2000 platform (2 × 100 bp paired-end reads). The remaining 152 libraries were prepared using the Illumina TrueSeq Stranded mRNA Kit, and sequenced on the Illumina HiSeq 2500 platform (2 × 125 bp, paired-end reads). Base calling was performed with the Illumina Real Time Analysis v1.18.61 software, and the Illumina Bc12fastq v1.8.4 was used for conversion to FastQ format. A total of 96 sequence files (741Gb) consisting of ~ 63 million short-reads per library were obtained from the HiSeq 2000 platform, and 1218 sequence files (~ 2 Tb) of ~ 23 million short-reads per library were obtained from the HiSeq 2500 platform. Eight samples were removed from further analysis due to low sequence quality, leaving a total of 168 samples for subsequent analyses. Sequence data has been deposited in the NCBI Sequence Read Archive accession number PRJNA403969.

Raw RNA sequence reads were first filtered for adapter sequences using Trimmomatic [73] followed by quality trimming using Condetri where the first six bases at the 3′ end and low quality reads were filtered out, retaining reads with a minimum length of 75 bases (Figure S5 in Additional file 2). The quality of each sequenced nucleotide was evaluated on adapter filtered and quality trimmed RNA-seq reads using the FASTX toolkit [74], and a mean Phred quality score of 37.01 ± 0.99 was obtained. After adapter and quality filtering, RNA-seq reads were mapped to the reference genome assembly *Sus scrofa* 11.1 using the splice aware aligner Tophat2 [75]. Sample-specific transcriptomes were assembled using Cufflinks and merged with the reference genome to create a set of known and novel isoforms using Cuffmerge [76]. A total of 28,033 full-length transfrags were identified (Figure S5 in Additional file 2). Alignment statistics and base coverage were obtained with SAMtools [77]. Samples showed on average 92.4% of sequencing reads mapping to the reference genome, and 73.3% were unique and properly paired with their complementary sequence (Figure S5 in Additional file 2). Total gene expression abundance was quantified using unique and properly paired reads using HTseq [78]. Genes with total count abundance less than 168 were removed from further analysis to reduce the number of genes with low expression, leaving 15,249 gene transcripts for eQTL analysis (Figure S5 in Additional file 2).

### RNA-seq count normalization and transformation

Expressed gene counts were normalized using the trimmed mean of M-values (TMM) to reduce systematic technical biases of sequenced transcripts [79]. TMM normalization has been shown to control false positive associations [80]. The normalized gene counts were then transformed to follow an approximately Gaussian distribution by calculating the log counts per million (log-cpm) as described in Law *et. al.* [81]. Briefly, a linear model was fit to obtain the expected log-cpm for each gene, *E*(*y*) = *xβ*, where y are the log-cpm, *x* is a vector of ones and *β* is a vector of estimated regression coefficients. The residual standard deviations for each gene and their calculated average log-cpm were used to estimate the mean variance trend, $\widehat{w}$, by fitting a LOWESS curve [81]. Variance coefficients were standardized to keep similar scales for residual variance and additive variance:

1 $$ \widehat{{\boldsymbol{w}}_{\boldsymbol{std}}}=\frac{\frac{1}{\sqrt{\widehat{w}}}}{\frac{1}{n}\sum \frac{1}{\sqrt{\widehat{\boldsymbol{w}}}}} $$

where, $\widehat{{\boldsymbol{w}}_{\boldsymbol{std}}}$ are the variance coefficients, *n* is the total number of animals, and $\widehat{\boldsymbol{w}}$ is the estimated mean variance trend. The normalized log-cpm were used as the response variable, ***y***, and the variance coefficients, $\widehat{{\boldsymbol{w}}_{\boldsymbol{std}}}$, were used to model heterogeneity of error variance in the eQTL scan. This approach accounts for the mean variance relationship of each gene expression instead of assuming equal variance for all observations.

### Heritability of phenotype and gene expression

A genomic best-linear unbiased prediction (GBLUP) model [70, 71] was used to estimate the heritability of each phenotype and gene expression by fitting the following equation:

2 $$ \boldsymbol{y}=X\boldsymbol{b}+\boldsymbol{a}+\boldsymbol{e}, $$

where, ***y*** is a vector with measurements of a phenotype for each animal when estimating phenotypic heritability, and a vector with normalized log-cpm gene expression when estimating the heritability of gene expression. *X* is an incidence matrix of fixed effects including sex and additional covariates unique to each phenotype [20, 21], and includes the transcriptional profiling selection scheme (i.e., within litter and sex extremes for loin muscle area or back fat thickness) when analyzing gene expression. The vector ***b*** contains the estimated fixed effect, ***a*** is a vector of random additive genetic effects and ***e*** is a vector of random residual errors. The additive genetic effects are assumed $\boldsymbol{a}\sim N\left(0\right.,G\left.{\sigma}_a^2\right)$ with the genomic relationship matrix [82], *G* ***=*** *ZZ*^′^. *Z* is a matrix of normalized SNP genotypes, with elements:

3 $$ Z=\frac{M-2p}{\sqrt{\sum \left(2\boldsymbol{p}\left(1-\boldsymbol{p}\right)\right)}}, $$

where, ***M*** is the matrix of SNP genotypes and ***p*** is a vector with the frequency of each reference allele. The error term is $\boldsymbol{e}\sim N\left(0,{\sigma}_e^2\ \mathit{\operatorname{diag}}\left(\widehat{{\boldsymbol{w}}_{\boldsymbol{std}}}\right)\ \right)$ with a variance inversely proportional to the variance coefficients, $\widehat{{\boldsymbol{w}}_{\boldsymbol{std}}}$. These variance coefficients account for the heteroskedasticity across genes with different expression. The heritability of gene expressions were calculated by taking the ratio of the variance of the additive genetic effects to the total phenotypic variance, ${h}^2={\sigma}_a^2/\left({\sigma}_a^2+{\sigma}_e^2\right)$.

Statistical significance of heritability was determined using a likelihood ratio test, $LR=2\left[ logL\left(\widehat{\theta}\right)- logL\left(\widehat{\theta_0}\right)\right],$comparing the likelihood of the model represented in Eq. 1 $\left(L\left(\widehat{\theta}\right)\right)$ and the likelihood of a null model that does not include the genetic additive effect $\left(L\left(\widehat{\theta_0}\right)\right)$. Testing the null hypothesis ${\sigma}_a^2=0$ is equivalent to testing *h*^2^ = 0. The likelihood ratios were compared to a chi-squared distribution with one degree of freedom, and the resulting *p*-value divided by 2 to account for the asymptotic distribution of the likelihood ratios that tend to follow a mixture of chi-square distributions with different degrees of freedom [83]. Multiple test corrections were performed using a FDR of 0.01 [84]. Differences in heritability between local and distant eQTL were determined with Wilcoxon rank sum test [85].

### Genome wide association

The SNP effects, $\widehat{\boldsymbol{g}}$**,** and their variances $Var\left(\widehat{\boldsymbol{g}}\right)$ were estimated as a linear transformation of the BLUP breeding values, $\widehat{\boldsymbol{a}}$, from Eq. 2 [86, 87]. A test statistic for the association of each marker with each phenotype or gene expression measure is computed by standardizing the SNP effects:

4 $$ \boldsymbol{T}=\frac{\widehat{\boldsymbol{g}}}{\sqrt{Var\left(\widehat{\boldsymbol{g}}\right)}}, $$

The *p*-values associated with this ***T*** test statistic were calculated using the Gaussian cumulative distribution function, Φ, as follows:

5 $$ p- value=2\left[1-\varPhi \left(\left|\boldsymbol{T}\right|\right)\right], $$

and subject to multiple test corrections per each gene (FDR ≤ 0.01) [84]. If a gene had more than one expressed transcript, the FDR was computed for the merged *p*-values of all transcripts expressed for the gene.

It has been demonstrated [86, 87] that the ***T*** test statistics and p-values resulting from Eqs. (4 and 5 are equivalent to those obtained from fitting a single marker model, specifically the Efficient Mixed-Model Association (EMMA) model [88].

### Local and distant regulators

Due to low SNP density and long-range LD in this pig population, distinguishing local versus distant regulation of gene expression is difficult. We applied the following algorithm to classify putative eQTL as local or distant regulators of a gene’s expression:

1. An eQTL was defined as any gene whose expression was associated with at least one marker surpassing the significance threshold (FDR ≤ 0.01).
2. The plausible position range of each eQTL was defined by the position of the first significant marker at the beginning of the QTL and last significant marker at the end of the QTL. If the eQTL had only one marker association, the position of the marker was used.
3. Given the mapped position of the gene profile (start and end position of the transcript), there are several possibilities
  - The associated eQTL plausible position range overlaps totally or partially: Local eQTL
  - The associated eQTL is on a different chromosome: Distant eQTL
  - The associated eQTL is on the same chromosome but does not overlap:
    i. There are non-significant SNP (FDR ≥ 0.01) between the mapped position of the gene profile and its associated eQTL range: Distant eQTL same chromosome
    ii. There are no SNP between gene and eQTL range (including the filtered SNP due to high LD): Plausible Local

The distance between an eQTL and the corresponding gene location was estimated as the difference between the peak SNP and the nearest position of the gene transcript.

### Co-localization analysis

The genomic positions of the mapped eQTL were co-localized with pQTL previously identified for the F2 MSUPRP for growth, carcass composition and meat quality traits. An eQTL was considered co-localized if its QTL position overlapped the mapped location of a pQTL. The statistical significance of each co-localized eQTL with pQTL was determined through a conditional analysis that tested the effect of the most significant marker associated with the pQTL on the co-localized eQTL gene expression, as follows:

6 $$ \boldsymbol{y}= Xb+{\boldsymbol{Z}}_{\boldsymbol{SNP}}{b}_{SNP}+\boldsymbol{a}+\boldsymbol{e}, $$

where, ***y*** is the expression of the co-localized eQTL gene. The *X****,b***, ***a*** *and* ***e*** were previously described in Eq. 2. ***Z***_***SNP***_ is a vector of standardized marker genotypes for the pQTL peak marker, co-localized with the eQTL gene, and *b*_*SNP*_ is the estimated marker effect. Type I error rate of 0.05 and Bonferroni p-value cutoff based on the number of tests performed (p-value ≤5.952e-04) was used to determine SNP effect significance. We also considered the effect the peak pQTL marker had on the eQTL peak by performing a linear transformation of the BLUP breeding values from Eq. 6 to estimate the individual SNP effects, and tested their significance as described in Eqs. (4 and 5. Multiple test corrections were performed using an FDR ≤ 0.01 [84]. If fitting the top pQTL marker completely eliminated the eQTL interval, the two QTL were considered to be significantly co-localized. The proportion of variance explained by the peak pQTL markers for each co-localized eQTL was estimated as described in Casiro et al. [21]. Briefly, the variance associated with the co-localized peak pQTL marker, ${\sigma}_{SNP}^2$, was estimated as:

7 $$ \widehat{\sigma_{SNP}^2}={b}^2\ \mathit{\operatorname{var}}\left({Z}_{SNP}\right), $$

where, *b*^2^ is the calculated peak pQTL marker effect from Eq. 6, and the proportion of gene expression variance accounted for by the co-localized pQTL peak SNP is $\widehat{\sigma_{SNP}^2}/\left(\widehat{\sigma_{SNP}^2}+\widehat{\sigma_a^2}+\widehat{\sigma_e^2}\right)$. The estimated additive genetic variance, ${\sigma}_a^2$, and error variance, ${\sigma}_e^2$, are obtained after fitting Eq. 6. A conditional analysis for each eQTL interval, fitting the peak SNP, was also performed to identify potential additional peaks within an eQTL. Pearson correlations among phenotypes and gene expressions were calculated using residuals from Eq. 6, and significance was determined with a t test and FDR ≤ 0.05. LD was estimated between the peak SNP for the pQTL and colocalized eQTL. Equations 6 and 7 were also used to estimate the proportion of gene expression variance explained by the *PRKAG3* T30 N SNP for all identified eQTL to uncover eQTL significantly associated with *PRKAG3,* and the proportion of phenotypic variance explained for meat quality phenotypes with an associated pQTL on SSC15.

### RT-qPCR

To verify the expression of *CHRNA9*, 28 animals were selected based on the genotypes of the peak eQTL SNP (10 animals per genotype equally weighted by sex except for the AA genotype that had only eight animals, four per sex). Total RNA was extracted from the longissimus muscle samples as described above, and 2 μg was reverse transcribed using the High Capacity cDNA Reverse Transcriptase Kit with RNase inhibitor (Applied Biosystems, Foster City, CA). A custom Taqman Gene Expression Assay (ThermoFisher Scientific, Waltham, MA) was designed for *CHRNA9* using pig RNA sequence to span exons 4 and 5 (determined based on the structure of the human *CHRNA9* gene, Accession No. AC118275). The GeNorm [89] algorithm was used to select two reference genes, *PPIA* (ThermoFisher Scientific Assay No. Ss03394781_g1) and *SDHA* (ThermoFisher Scientific Assay No. Ss03376909_u1), with the highest gene-stabilizing measure to normalize the expression of *CHRNA9.* RT-qPCR was performed in triplicate using 50 ng cDNA and TaqMan Gene Expression Master Mix for a final volume of 20 μl. Assays were run on a StepOnePlus Real-Time PCR System (Applied Biosystems). The cycling conditions were 52 °C for 2 min, 95 °C for 10 min followed by 50 cycles of 95 °C for 15 s and 60 °C for 1 s. ∆Ct values were calculated as the mean difference between the geometric mean of the reference genes and the target gene. To verify the RNA-seq results, the effect of the peak eQTL marker for *CHRNA9* was measured using Eq. 6 with the response variable being the ∆Ct transcript abundance. Analysis of variance with a type I error rate of 0.05 was used to determine significant additive and dominance effects of the peak *CHRNA9* eQTL SNP (DIAS0000678).

## Additional files

Additional file 1: **Table S1.** Expression quantitative trait loci (eQTL) mapped for *longissimus dorsi* muscle transcripts from the MSUPRP (*n* = 168). **Table S2.** Phenotypic QTL identified in the F2 MSUPRP. **Table S3.** Results of conditional analysis for expression QTL co-localized with phenotypic QTL. **Table S4.** Pearson correlations of genes associated with the H3GA0052416 marker on SSC15 and protein percent. **Table S5.** Conditional analysis: PRKAG3 SNP effect on eQTL gene’s expression. **Table S6.** Comparison of RT-qPCR and RNA-seq *CHRNA9* gene expression association with SNP DIAS0000678. **Table S7.** Summary statistics for phenotypic traits for the MSUPRP and the subsample used in this study. (XLSX 200 kb)

Additional file 2: **Figure S1.** Manhattan plots illustrating classification of different types of gene expression regulation based on eQTL position. **Figure S2.** Heritability of transcript profiles. **Figure S3.** Pearson correlations among phenotypic traits with an associated pQTL. **Figure S4.** Proportion of phenotypic variance explained by PRKAG3 and H3GA0052416 SNP for meat quality traits. **Figure S5.** RNA-seq pipeline. (PDF 2134 kb)

## Abbreviations

CEP128: Centrosomal protein 128
CHRNA9: Cholinergic receptor nicotinic alpha 9 subunit
CIT: Citron Rho-interacting serine/threonine kinase
eQTL: Expression quantitative trait locus
FDR: False discover rate
FRMD8: FERM domain-containing 8 gene
GBLUP: Genomic best-linear unbiased prediction
GWAS: Genome wide association study
h^2^: Heritability
LD: Linkage disequilibrium
MRLN: Myoregulin
MSUPRP: Michigan State University Pig Resource Population
NQO1: NADPH quinone oxidoreductase − 1
PFKFB3: 6-phosphofructo-2-kinase / fructose-2-,6-biphosphatase 3
PI3K-Akt-mTOR: Phosphatidylinositol-3-kinase/Akt/mammalian target of rapamycin
PKP2: Plakophilin
pQTL: Phenotypic quantitative trait locus
PRKAG3: Protein kinase AMP-activated non-catalytic subunit gamma3
QTL: Quantitative trait locus
SNP: Single nucleotide polymorphism
SSC: *Sus scrofa* chromosome
SUCLG2: Succinate-COA ligase GDP-forming beta subunit
TMM: Trimmed mean M-values
WBS: Warner Bratzler shear force
