# Supplementary material for: Genetic control of longissimus dorsi muscle gene expression variation and joint analysis with phenotypic quantitative trait loci in pigs
Source: BMC Genomics. 2019 Jan 3;20:3. doi: 10.1186/s12864-018-5386-2 (PMC6319002; doi:10.1186/s12864-018-5386-2)
Supplement: Supplementary file 2 — Figure S1. Manhattan plots illustrating classification of different types of gene expression regulation based on eQTL position. Figure S2. Heritability of transcript profiles. Figure S3. Pearson correlations among phenotypic traits with an associated pQTL. Figure S4. Proportion of phenotypic variance explained by PRKAG3 and H3GA0052416 SNP for meat quality traits. Figure S5. RNA-seq pipeline. (PDF 2134 kb) [file 12864_2018_5386_MOESM2_ESM.pdf]

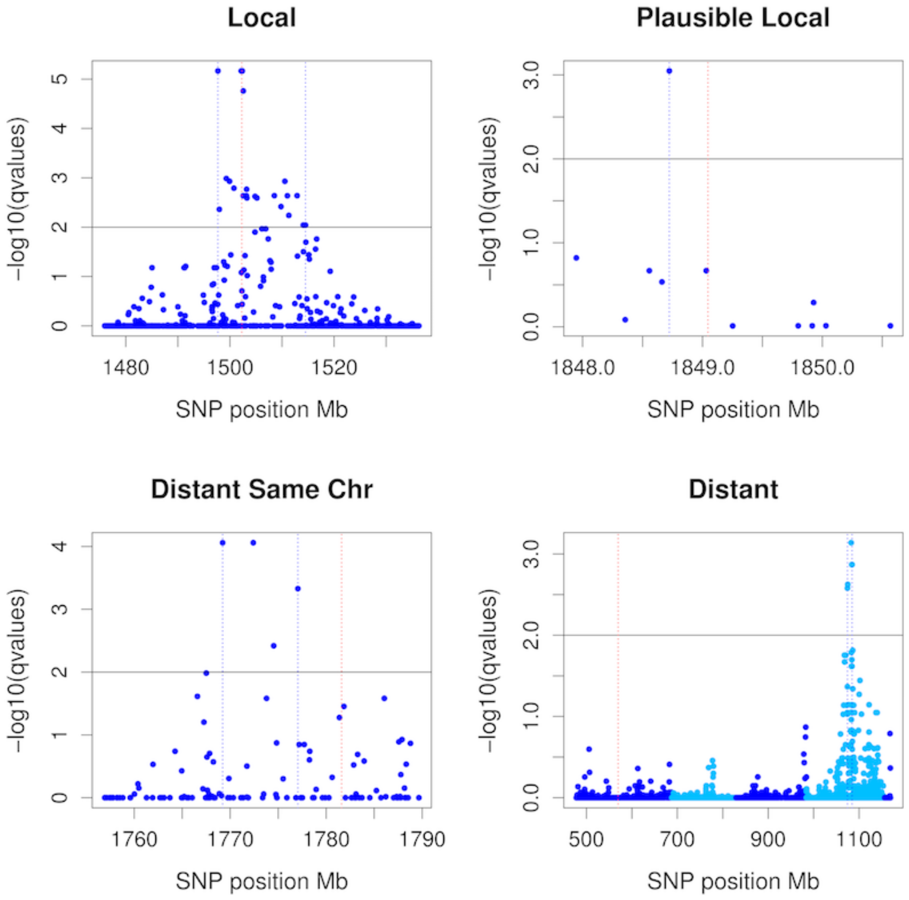

Figure S1. Manhattan plots illustrating classification of different types of gene expression regulation based on eQTL position. The x-axis represents the absolute genomic position of the marker and the y-axis the significance of the association with the gene transcript, -log10 q-value. The two blue vertical dotted lines delimit the eQTL plausible position range (eQTL-PPR), and the vertical red dotted line indicates the absolute position of the gene transcript. Local-acting regulator: the position of the gene transcript falls within or overlaps the eQTL-PPR. Plausible local regulator: the eQTL-PPR does not contain or overlap the gene transcript and the density of SNP in the region separating the two is zero. Distant-acting regulator on the same chromosome:

the position of the gene transcript falls outside the specified eQTL-PPR but on the same chromosome and the SNPs between the genomic position of the gene and the eQTL-PPR do not surpassing the significance threshold. Distant-acting regulator: the eQTL-PPR is on a different chromosome than the genomic position of the associated gene transcript.

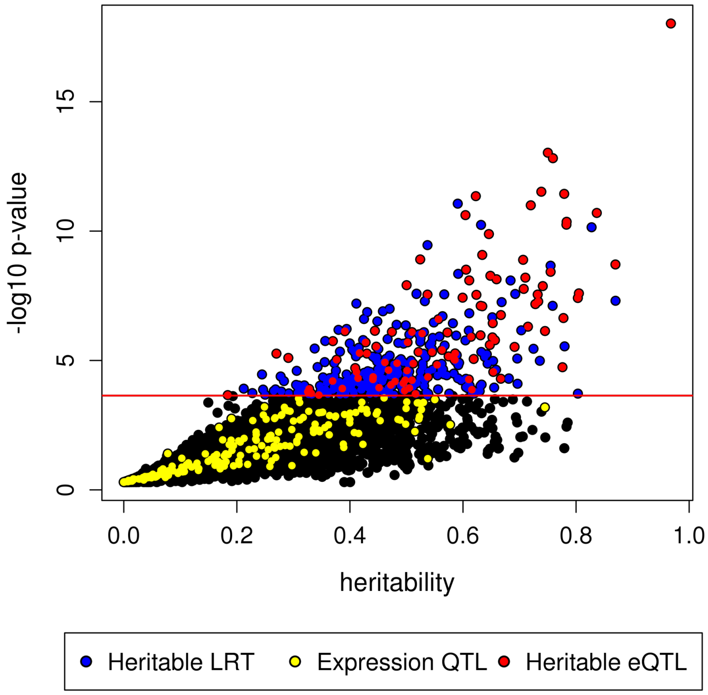

Figure S2. Heritability of transcript profiles. Heritability of genes is shown on the x-axis and p-values from the likelihood ratio test (LRT) for significant heritable expression are on the y-axis. A total of 344 gene expression transcripts were found to be heritable (shown in blue and red, FDR ≤ 0.01). A significant enrichment of genes with associated eQTL was observed among the heritable genes (103 genes; p-value ≤ 2.2e-16; shown in red). The 218 genes associated with an eQTL that did not surpass the threshold for significant heritability are shown in yellow.

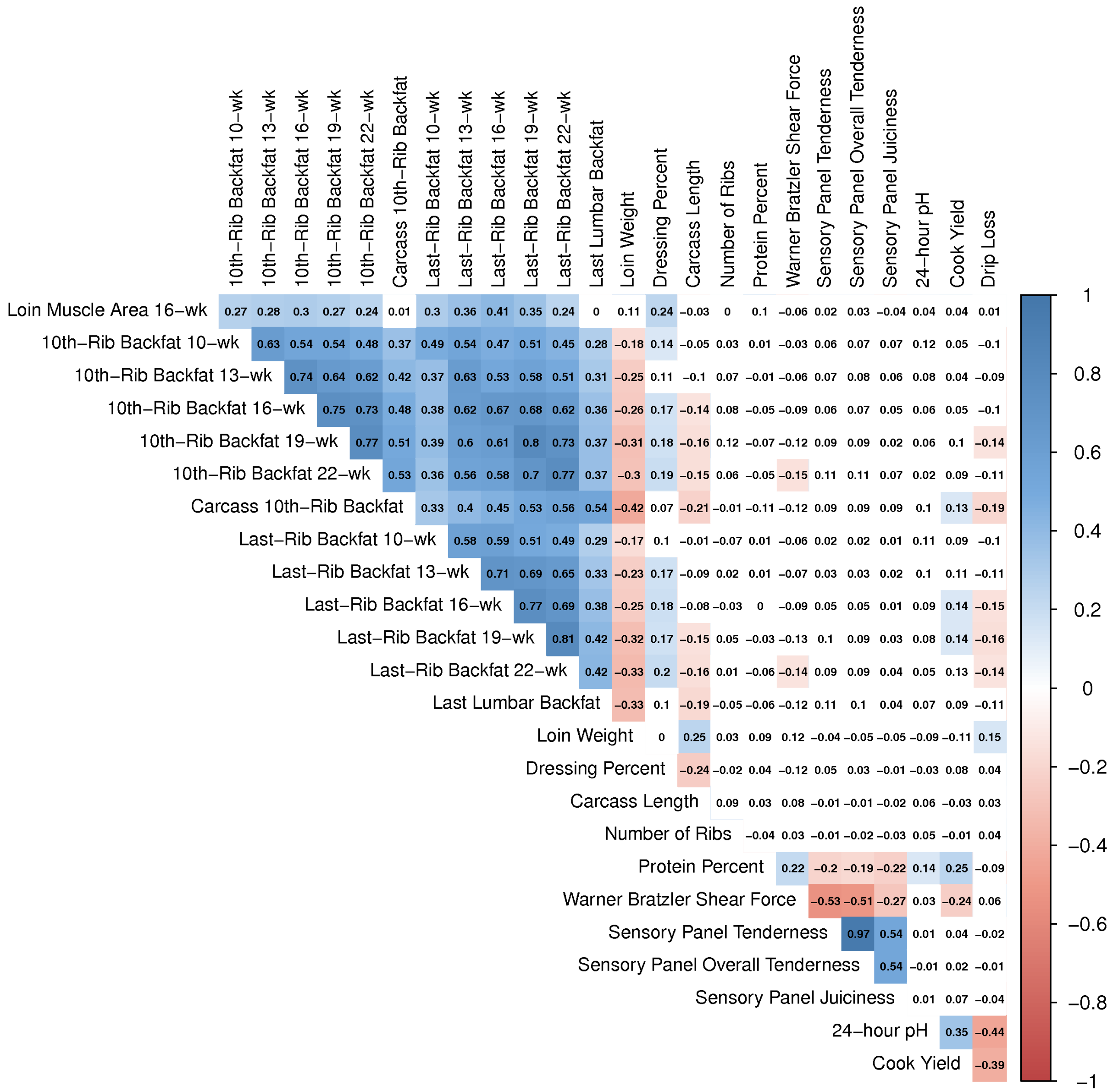

Figure S3. Pearson correlations among phenotypic traits with an associated pQTL. Significant correlations are shaded in color, p-value ≤ 8e-05, with shades of red depicting negative correlations and shades of blue depicting positive correlations.

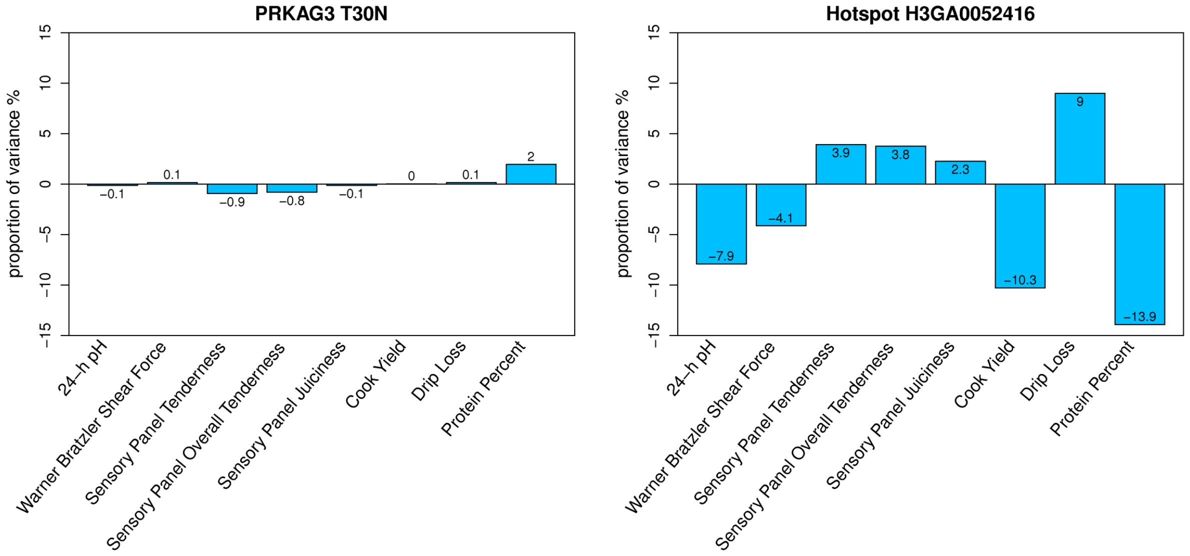

Figure S4. Proportion of phenotypic variance explained by PRKAG3 and H3GA0052416 SNP for meat quality traits. Traits are shown on the x-axis, and the proportion of phenotypic variance explained by the SNP marker is shown on the y-axis. Directionality of bar plots indicates the SNP effect on the phenotype. The H3GA0052416 marker was considered a plausible putative hotspot on SSC15.

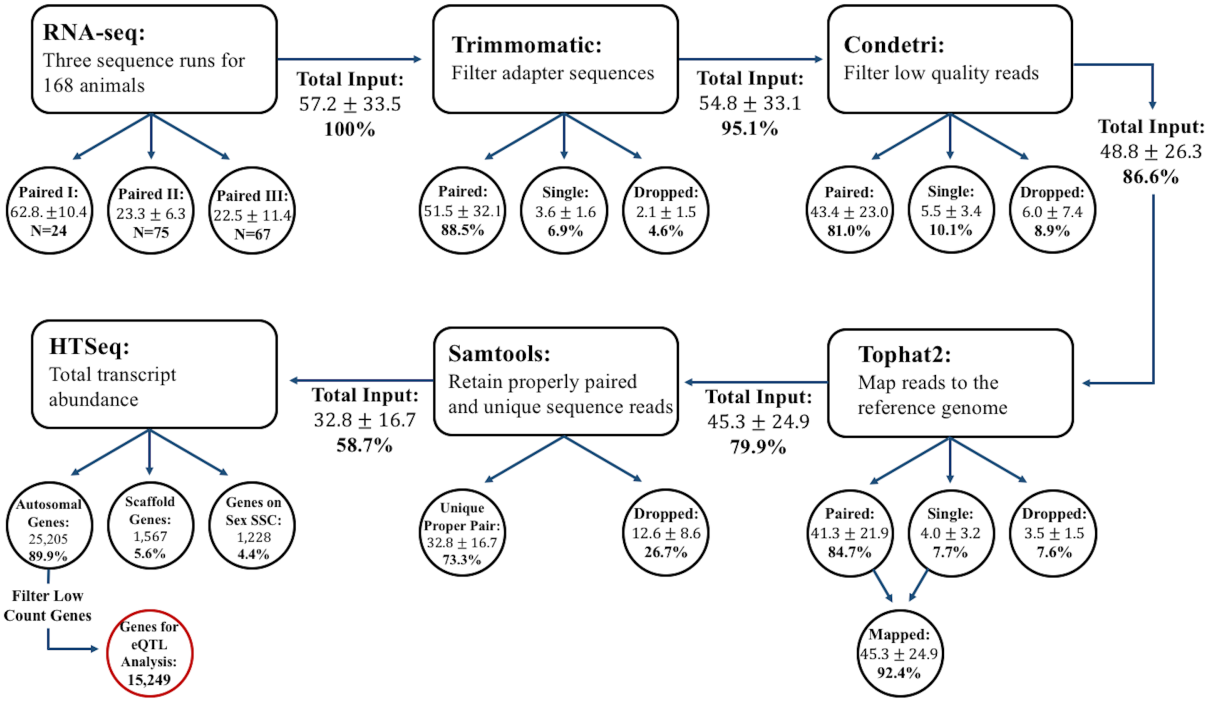

Figure S5. RNA-seq pipeline. Bioinformatic pipeline used in this study with summary statistics.
